# Paraoxonase 1 (PON1) Polymorphisms, Haplotypes and Activity in Predicting CAD Risk in North-West Indian Punjabis

**DOI:** 10.1371/journal.pone.0017805

**Published:** 2011-05-24

**Authors:** Nidhi Gupta, Surjit Singh, V. Nagarjuna Maturu, Yash Paul Sharma, Kiran Dip Gill

**Affiliations:** 1 Department of Biochemistry, Post Graduate Institute of Medical Education and Research (PGIMER), Chandigarh, India; 2 Department of Internal Medicine, Post Graduate Institute of Medical Education and Research (PGIMER), Chandigarh, India; 3 Department of Cardiology, Post Graduate Institute of Medical Education and Research (PGIMER), Chandigarh, India; Dr. Margarete Fischer-Bosch Institute of Clinical Pharmacology, Germany

## Abstract

**Background:**

Human serum paraoxonase-1 (PON1) prevents oxidation of low density lipoprotein cholesterol (LDL-C) and hydrolyzes the oxidized form, therefore preventing the development of atherosclerosis. The polymorphisms of PON1 gene are known to affect the PON1 activity and thereby coronary artery disease (CAD) risk. As studies are lacking in North-West Indian Punjabi's, a distinct ethnic group with high incidence of CAD, we determined PON1 activity, genotypes and haplotypes in this population and correlated them with the risk of CAD.

**Methodology/Principal Findings:**

350 angiographically proven (≥70% stenosis) CAD patients and 300 healthy controls were investigated. PON1 activity was determined towards paraoxon (Paraoxonase; PONase) and phenylacetate (Arylesterase; AREase) substrates. In addition, genotyping was carried out by using multiplex PCR, allele specific oligonucleotide –PCR and PCR-RFLP methods and haplotyping was determined by PHASE software. The serum PONase and AREase activities were significantly lower in CAD patients as compared to the controls. All studied polymorphisms except L55M had significant effect on PONase activity. However AREase activity was not affected by them. In a logistic regression model, after adjustment for the conventional risk factors for CAD, QR (OR: 2.73 (1.57–4.72)) and RR (OR, 16.24 (6.41–41.14)) genotypes of Q192R polymorphism and GG (OR: 2.07 (1.02–4.21)) genotype of −162A/G polymorphism had significantly higher CAD risk. Haplotypes L-T-G-Q-C (OR: 3.25 (1.72–6.16)) and L-T-G-R-G (OR: 2.82 (1.01–7.80)) were also significantly associated with CAD.

**Conclusions:**

In conclusion this study shows that CAD patients had lower PONase and AREase activities as compared to the controls. The coding Q192R polymorphism, promoter −162A/G polymorphism and L-T-G-Q-C and L-T-G-R-G haplotypes are all independently associated with CAD.

## Introduction

The oxidative modification of low-density lipoprotein cholesterol (LDL-C) in the arterial wall is believed to be the major pathogenetic mechanism behind the initiation and acceleration of atherosclerosis, and thus the coronary artery disease (CAD) [1]. High-density lipoprotein cholesterol (HDL-C), on the other hand is known to have a protective effect. The protective role of HDL-C is believed to be mainly due to enzyme paraoxonase 1 (PON1), a Ca^++^ dependent esterase, bound to its surface which probably prevents atherosclerosis by preventing LDL-C from peroxidation [2]. In addition to preventing peroxidation, it also hydrolyzes the oxidized LDL as shown *in-vivo* and *in-vitro* studies [3], [4]. It has been shown that PON1 knockout mice cannot hydrolyze the oxidized LDL and have increased risk of developing CAD [5].

There exists a wide variation in PON1 activity in different ethnic groups and within individuals in the same ethnic group [6]. The PON1 activity has been shown to be lower after acute myocardial infarction [7]. It is also lower in patients with familial hypercholesterolemia and diabetes mellitus, who are more prone to CAD [8]. This has led to the hypothesis that the lower the PON1 activity is, the higher will be the accumulation of oxidized LDL and risk of CAD.

The PON1 gene has nearly 200 SNPs (single nucleotide polymorphisms) [9]. Of these −909G/C [rs854572], −162A/G [rs705381], −108C/T [rs705379] polymorphisms located in the promoter and Q192R [rs662] and L55M [rs 854560] polymorphisms located in the coding region are the most commonly studied SNPs. PON1 hydrolyzes a variety of organophosphates including paraoxon (PONase), diazoxon (DZOase), sarin and soman and arylesters such as phenylacetate (AREase) [10]. Following purification of rabbit [11] and human [12] PON1, it was confirmed that it can hydrolyze both paraoxon and phenylacetate [13]. PON1 Q192R polymorphism affects the catalytic efficiency of PON1 towards some of its substrates [14]. The PON1 R192 isoform hydrolyses paraoxon rapidly as compared to PON1 Q192 isoform, whereas Q192 isoform hydrolyses diazoxon, sarin and soman rapidly as compared to R192 isoform [15]. However, phenylacetate hydrolysis i.e. AREase activity is not affected by Q192R polymorphism and has been shown to correspond with PON1 levels [9], [16], [17] determined by immunological methods [18].

There are several studies demonstrating an association between Q192R and L55M polymorphisms and susceptibility to CAD [19], [20]. However others have failed to find such an association [21], [22], [23]. A few studies have also looked at association between −108C/T polymorphism and risk of CAD [24]. Literature is almost silent regarding the effect of −909G/C, −162A/G polymorphisms and PON1 haplotypes on CAD risk.

The North West Indian Punjabi's are considered to be a progeny of many proto and post Harappan invaders who entered lands from West and became original settlers of the area. They are thought to be progeny of Indo – Scythian, Indo European and Indo – Aryan stocks [25]. Singh et al. [26] have shown that healthy North-West Indian Punjabis have lower PONase activity as compared to Caucasian whites. In a subsequent study, in same population it was shown that CAD patients with or without type II diabetes mellitus have lower PONase activity as compared to healthy controls [27]. However, information regarding PON1 coding and promoter polymorphisms in this population is not available. In the present case-control study we investigated the effect of PON1 polymorphisms on PON1 activity and lipid levels in angiographically proven CAD patients. Further, we also investigated the association of PON1 genotypes and haplotypes with the risk of CAD in the same population.

## Results

### Baseline characteristics of the study population

In the CAD group, 180 patients had a single vessel involved whereas 190 had double and 52 had triple vessel disease. Compared to the controls, CAD group had greater proportion of men, alcohol consumers and were significantly older, had higher BMI and lower serum HDL-C levels (p = 0.0001) (Table 1). However no significant difference was observed in LDL-C, TG and TC (p>0.05) between the groups (Table 1).

**Table 1:** Demographic, clinical and biochemical characteristics of Controls and CAD patients.

Variables	Controls (n = 300)	CAD (n = 350)
Age (yrs)	43.1±10.7	55.9±9.7*
Male/Female (n)	151/149	286/64*
BMI (kg/m^2^)	23.5±4.1	27.1±3.9*
SBP (mm Hg)	120.9±10.7	129±15.4*
DBP (mm Hg)	80±6.9	85±7.7*
Alcohol consumers	18 (6.0%)	86 (24.6%)
Non consumers	282 (94.0%)	264* (75.4%)
Smokers	50 (16.7%)	73 (20.9%)
Non-Smokers	250 (83.3%)	277 (79.1%)
HDL-C, mmol/L	1.23±0.20	1.08±0.26*
LDL-C, mmol/L	2.30±0.51	2.37±0.89
TG, mmol/L	1.53±0.63	1.50±0.57
TC, mmol/L	4.32±1.08	4.43±1.27
PONase activity (nmol/min/ml)	178.0 (28.5–410.0)	113.0* (21.30–307.5)
AREase activity (µmol/min/ml)	82.7 (22.8–293.0)	74.6* (15.2–149.0)
HDL-C/PONase ratio	7.8×10^−3^±4×10^−3^	12.2*×10^−3^±5.6×10^−3^
Number of diseased vessels		
Single vessel, n (%)	------	108 (30.9%)
Double vessel, n (%)	------	190 (54.4%)
Triple vessel, n (%)	------	52 (14.6%)

Values are mean ± SD or median (range).

*P<0.0001 vs. controls.

BMI: body mass index; TG: triglycerides; TC: total cholesterol; PONase: Paraoxonase; AREase: Arylesterase.

### Serum PON1 activity in the study population

The CAD patients had significantly lower serum PONase (113.0 nmol/min/ml vs. 178.0 nmol/min/ml) and AREase activities (74.6 µmol/min/ml vs. 82.7 µmol/min/ml) (P<0.0001) as compared to the controls (Table 1). The HDL/PONase ratio was significantly higher in patients (Table 1). The differences in PON1 activity (PONase and AREase) between the controls and patients was tested for independence from other variables by a multiple linear regression analysis. The model included age, BMI, sex, smoking, alcohol consumption, HDL-C, LDL-C, TG, TC, Q192R, −909 G/C, −162A/G and −108C/T polymorphisms. The difference in PONase activity was found to be dependent on differences in BMI (P = 0.0001), coding Q192R (P = 0.0001), promoter −909G/C (P = 0.0001), −162A/G (P = 0.0001) and −108C/T (P = 0.001) polymorphisms (Table 2). However AREase activity was found to be independent of these polymorphisms (P>0.05) (data not shown).

**Table 2:** Multiple linear regression analysis for PONase activity.

Variables	Unstandardized coefficients		Standardized coefficient (β)	t	P value
	B	Std. Error			
Age	−0.016	0.217	−0.003	−0.074	0.941
Sex	3.060	5.165	0.019	0.592	0.554
Q192R	34.140	3.349	0.310	10.193	0.000
−909G/C	−15.753	3.100	−0.157	−5.082	0.000
−162A/G	−19.797	3.186	−0.191	−6.214	0.000
−108C/T	−19.646	3.167	−0.191	−6.204	0.000
Smoking status	.941	6.053	0.005	0.156	0.876
Alcohol status	3.843	6.593	0.019	0.583	0.560
BMI	−2.187	0.578	−0.123	−3.781	0.000
HDL-C	0.176	0.242	0.023	0.728	0.467
LDL-C	−0.096	0.080	−0.037	−1.205	0.229
TG	0.086	0.044	0.061	1.975	0.06
TC	0.012	0.050	0.008	0.248	0.804
Groups	−53.498	6.243	−0.357	−8.570	0.000

BMI: body mass index; TG: triglycerides; TC: total cholesterol.

### Effect of PON1 polymorphisms on PON1 activity

**Figure 1:**
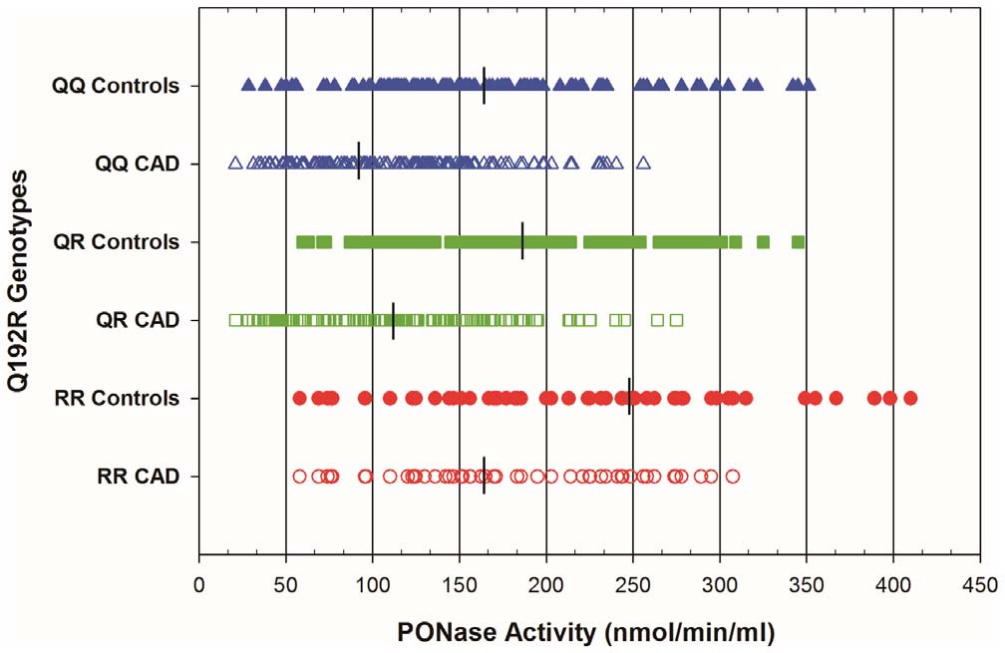
Individual data point for PONase activity in Controls and CAD patients for each PON1 Q192R genotype. Medians are indicated by crossbars.

**Figure 2:**
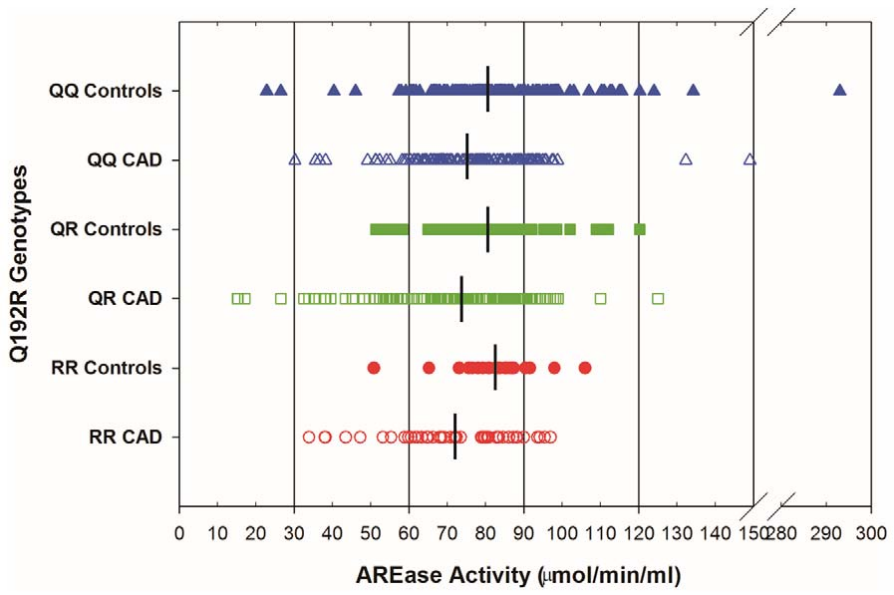
Individual data point for AREase activity in Controls and CAD patients for each PON1 Q192R genotype. Medians are indicated by crossbars.

Figure 1 shows the effect of the PON1 Q192R polymorphism on PONase activity across Q192R genotypes in controls and CAD patients. The PONase activity in patients ranged from 21.30 to 307.5 nmol/min/ml and from 28.5 to 410.0 nmol/min/ml in controls. PONase activity was significantly lower in QQ homozygotes followed by QR heterozygotes and RR homozygotes (QQ<QR<RR) in patients (95.0<113.0<165.0 nmol/min/ml) and controls (165.0<189.0<248.4 nmol/min/ml) (Fig. 1). It provides an excellent example of why substrates whose rates of hydrolysis are affected by the PON1 Q192R polymorphism should not be used to compare levels across genotypes. Figure 2 shows PON1 levels estimated by AREase activity across Q192R genotypes for controls and CAD patients. The AREase activity in controls ranged from 22.8 to 293.0 µmol/min/ml and, in patients from 15.28 to 149.0 µmol/min/ml. The median PON1 levels, were similar across Q192R genotypes for controls (QQ = 82.63, QR = 82.59 and RR = 83.04 µmol/min/ml) and CAD patients (QQ = 76.21, QR = 71.52 and RR = 70.16 µmol/min/ml).

L55M polymorphism had no effect on serum PONase and AREase activities in both the groups. However when comparison was carried out between groups within genotypes, both activities was significantly lower in LL, LM and MM genotypes in CAD patients as compared to the controls (Table 3). PONase activity was affected by promoter −909G/C, −162A/G and −108C/T polymorphisms in both controls and patients (Table 3). It was highest in wild GG (−909G/C), AA (−162A/G) and CC (−108C/T) homozygotes and lowest in variant CC (−909G/C), GG (−162A/G) and TT (−108C/T) homozygotes, whereas heterozygotes GC (−909G/C), AG (−162A/G) and CT (−108C/T) had intermediate PONase activity (Table 3). However, AREase activity was not affected by any of these promoter polymorphisms (Table 3). When comparison was carried out between groups within genotypes, patients had lower PONase and AREase activities in all genotypes as compared to the controls (Table 3).

**Table 3:** PONase and AREase activities in Controls and CAD patients according to their coding L55M and promoter −909G/C, −162A/G, and −108C/T polymorphisms.

L55M	Controls			CAD		
	LL	LM	MM	LL	LM	MM
PONase activity (nmol/min/ml)	186.0(47.4–410.0)	156.0(28.5–398.0)	150.0(123.0–231.0)	111.0§(21.0–307.5)	116.0§(21.0–256.0)	85.65∥(64.5–93.0)
AREase activity (nmol/min/ml)	82.8(22.8–293.0)	81.7(51.3–134.2)	83.5(61.0–107.0)	76.1§(17.07–149.0)	70.7§(15.20–132.3)	75.8(61.22–88.6)
**−909G/C**	**GG**	**GC**	**CC**	**GG**	**GC**	**CC**
PONase activity (nmol/min/ml)	195.0(47.4–389.0)	174.0(59.6–410.0)	163.8*(28.5–398.0)	134.7§(21.0–307.5)	123.2§(21.0–307.5)	74.4* §(21.0–264.0)
AREase activity (nmol/min/ml)	84.4(22.8–134.2)	82.4(22.9–293.0)	81.1(40.3–115.6)	76.1§(15.2–132.3)	73.0§(15.2–125.0)	75.4∥(17.0–149.0)
**−162A/G**	**AA**	**AG**	**GG**	**AA**	**AG**	**GG**
PONase activity (nmol/min/ml)	198.0(59.6–410.0)	178.0(28.5–389.0)	160.5†(38.2–398.0)	146.5§(35.6–295.0)	123.0§(21.0–307.5)	88.0† §(21.0–274.7)
AREase activity (nmol/min/ml)	82.6(22.8–293.0)	82.67(46.0–115.6)	83.0(40.3–112.5)	71.4§(38.2–149.0)	75.3§(15.2–132.3)	76.1§(15.2–97.6)
**−108C/T**	**CC**	**CT**	**TT**	**CC**	**CT**	**TT**
PONase activity (nmol/min/ml)	214.5(53.3–410.0)	170.5(50.1–367.0)	144.5‡(28.5–398.0)	142.5§(37.4–307.5)	112.6§(21.0–248.5)	86.7‡ §(21.0–289.2)
AREase activity (nmol/min/ml)	80.8(22.8–293.0)	83.4(55.8–134.23)	83.0(51.3–115.0)	78.0∥(33.9–110.0)	75.6§(15.2–149.0)	71.3§(15.27–98.79)

Values are median (range).

* Significantly different from GG+GC genotype P<0.0001;

† Significantly different from AA+GG genotype p<0.0001;

‡ Significantly different from CC+CT genotype P<0.0001;

§ Significantly different from controls P<0.0001;

∥ Significantly different from controls P<0.05.

### Effect of PON1 polymorphisms on lipid levels

HDL-C was significantly lower (P<0.05) in QQ (Q192R), and GG (−162A/G) genotypes and TG was significantly higher (P<0.05) in GG (−909G/C) and RR (Q192R) genotypes in patients as compared to controls. No significant difference was observed for LDL-C and TC in any of the group (data not shown).

### Association of coding Q192R and L55M polymorphisms with CAD

The genotype and allele frequency distribution of coding Q192R and L55M polymorphisms of CAD patients and controls is shown in Table 4. All the studied polymorphisms in patients and controls followed Hardy–Weinberg equilibrium (HWE) (P>0.05). In Q192R polymorphism, QR and RR genotypes were significantly associated with CAD (P = 0.0001) with ORs being 2.08 (95% CI: 1.49–2.90) and 2.92 (95% CI: 1.71–4.98) respectively as compared to wild QQ homozygotes. The frequency of minor R allele in patients was significantly higher (0.39) in patients as compared to that in controls (0.26) (OR: 2.23; 95% CI: 1.61–3.06). In L55M polymorphism, genotype and allele frequency distribution was not significantly different (P>0.05) between the groups.

**Table 4:** PON1 coding (Q192R, L55M) and promoter (−909G/C, −162A/G and −108C/T) genotypes and allele frequencies distribution in Controls and CAD patients.

SNPs	Controls	CAD	OR	95% CI	P value
Q192R genotypes					
QQ*	168 (56%)	127 (36.3%)	1 .00	-	-
QR	108 (36%)	170 (48.6%)	2.08	1.49–2.90	0.0001
RR	24 (8.0%)	53 (15.1%)	2.92	1.71–4.98	0.0001
Allele frequency					
Q*	0.74	0.61	1.00		
R	0.26	0.39	2.23	1.61–3.06	0.0001
L55M genotypes					
LL	193 (64.3%)	247 (70.6%)	1.92	0.53–6.89	0.31
LM	101 (33.7%)	99 (28.3%)	1.47	0.40–5.36	0.56
MM*	6 (2.0%)	4 (1.1%)	1.00	-	
Allele frequency					
L	0.81	0.85	1.76	0.49–6.31	0.38
M*	0.19	0.15	1.00		
−909G/C genotypes					
GG*	118 (39.3%)	80 (22.9%)	1.00	-	
GC	129 (43%)	160 (45.7%)	1.80	1.24–2.59	0.002
CC	53 (17.7%)	110 (31.4%)	3.03	1.96–4.68	0.0001
Allele frequency					
G*	0.61	0.46	1.00		
C	0.39	0.54	2.15	1.53–3.03	0.0001
−162A/G genotypes					
AA*	97 (32.3%)	56 (16.0%)	1.00		
AG	141 (47%)	171 (48.9%)	2.12	1.41–3.12	0.0001
GG	62 (20.7%)	123 (35.1%)	3.43	2.19–5.38	0.0001
Allele frequency					
A*	0.56	0.40	1.00		
G	0.44	0.60	2.50	1.72–3.64	0.0001
−108C/T genotypes					
CC*	100 (33.3%)	68 (19.4%)	1.00		
CT	140 (46.7%)	166 (47.4%)	1.74	1.19–2.55	0.001
TT	60 (20%)	116 (33.1%)	2.84	1.83–4.40	0.0001
Allele frequency					
C*	0.57	0.43	1.00		
T	0.43	0.57	2.07	1.45–2.90	0.0001

*Reference Category; OR: odds ratio; CI: confidence interval.

Association between Q192R polymorphism and CAD was followed up with multiple logistic regression to determine if the association was independent of other known risk factors for CAD, such as alcohol consumption, HDL-C levels, age, and sex. In the regression analysis, age, sex, alcohol consumption, HDL-C levels and BMI were associated with CAD. The association between Q192R polymorphism and CAD persisted in this analysis. The ORs were 2.73 (95% CI: 1.57–4.72) and 16.24 (95% CI: 6.41–41.14) for QR heterozygote and RR homozygote respectively (Table 5).

**Table 5:** Multiple logistic regression analysis of Q192R polymorphism and association with CAD.

Variables	B	S.E.	OR	95% CI	P value
Age	0.11	0.01	1.11	1.08–1.14	0.0001
Sex-Female*			1.000	-	-
Male	1.08	0.27	2.95	1.70–5.10	0.0001
PONase activity	−0.01	0.002	0.98	0.97–0.98	0.0001
AREase activity	−0.02	0.009	0.97	0.96–0.99	0.001
Alcohol consumer-never*			1.000		
Current	1.32	0.39	3.77	1.74–8.12	0.001
SBP	0.04	0.01	1.04	1.02–1.07	0.001
HDL-C	−0.06	0.01	0.94	0.91–0.96	0.0001
BMI	0.11	0.03	1.12	1.05–1.20	0.0001
Q192R-QQ*			1.00	-	-
QR	1.00	0.28	2.73	1.57–4.72	0.0001
RR	2.78	0.47	16.24	6.41–41.14	0.0001

*Reference Category; OR: odds ratio; CI: confidence interval. BMI: body mass index; TG: triglycerides; TC: total cholesterol; PONase: Paraoxonase; AREase: Arylesterase.

### Association of Promoter −909G/C, −162A/G and −108C/T polymorphisms with CAD

In promoter −909G/C polymorphism, GC and CC genotypes were significantly associated with CAD (P = 0.0001) with ORs being 1.80 (95% CI: 1.24–2.59) and 3.03 (95% CI: 1.96–4.68) respectively (Table 4) as compared to wild GG homozygote. The frequency of minor C allele in patients was significantly higher (0.54) as compared to that in controls (0.39) (OR: 2.15; 95% CI: 1.53–3.03). In −162A/G polymorphism, AG and GG genotypes were significantly associated with CAD (P = 0.0001) with ORs being 2.12 (95% CI: 1.41–3.12) and 3.43 (95% CI: 2.19–5.38) respectively (Table 4) as compared to wild AA homozygote. The frequency of minor G allele in patients was significantly higher (0.60) as compared to that in controls (0.44) (OR: 2.50; 95%CI: 1.72–3.64). In −108C/T polymorphism, CT and TT genotypes were also significantly associated with CAD (P = 0.001) with ORs being 1.74 (95% CI: 1.19–2.55) and 2.84 (95% CI: 1.83–4.40) respectively (Table 4) as compared to wild CC homozygote. The frequency of minor T allele in patients was significantly higher (0.57) as compared to that in controls (0.43) (OR: 2.07; 95% CI: 1.45–2.90).

However after multiple logistic regression analysis in which cardiovascular risk factors were adjusted, the association was observed only with −162A/G polymorphism and CAD with OR being 2.07 (95% CI: 1.02–4.21) for GG homozygotes (Table 6). No significant association was observed with other two promoter polymorphisms (−909G/C and −108C/T) (P>0.05) (data not shown).

**Table 6:** Multiple logistic regression analysis of −162 A/G polymorphism and association with CAD.

Variables	B	S.E.	OR	95% CI	P value
Age	0.10	0.01	1.11	1.08–1.13	0.0001
Sex-Female*			1.000	-	-
Male	1.06	0.26	2.90	1.72–4.89	0.0001
PONase activity	−0.01	0.002	0.98	0.98–0.99	0.0001
AREase activity	−0.02	0.008	0.97	0.95–0.99	0.001
Alcohol consumer-never*			1.000		
Current	1.45	0.38	4.29	2.02–9.08	0.001
SBP	0.04	0.01	1.04	1.02–1.07	0.001
HDL-C	−0.06	0.01	0.93	0.91–0.96	0.0001
BMI	0.11	0.03	1.12	1.05–1.20	0.0001
−162A/G-AA*			1.00	-	-
AG	0.54	0.31	1.72	0.93–3.19	0.08
GG	0.73	0.36	2.07	1.02–4.21	0.04

*Reference Category; OR: odds ratio; CI: confidence interval. BMI: body mass index; TG: triglycerides; TC: total cholesterol; PONase: Paraoxonase; AREase: Arylesterase.

### Linkage disequilibrium (LD) analysis

LD values were generated to look for association among the five polymorphisms. No significant LD was observed among the PON1 SNPs with D' value ranging from 0.03 to 0.25 (Table 7).

**Table 7:** Pair wise comparison of measures of LD (D') for the polymorphisms of PON1 gene.

Variant 1	Variant 2	D'
−909G/C (rs 854572)	−162A/G (rs 705381)	0.21
−909G/C (rs 854572)	−108 C/T (rs 705379)	0.22
−909G/C (rs 854572)	L55M (rs 854560)	0.09
−909G/C (rs 854572)	Q192R (rs 662)	0.03
−162A/G (rs 705381)	−108 C/T (rs 705379)	0.15
−162A/G (rs 705381)	L55M (rs 854560)	0.10
−162A/G (rs 705381)	Q192R (rs 662)	0.10
−108 C/T (rs 705379)	L55M (rs 854560)	0.03
−108 C/T (rs 705379)	Q192R (rs 662)	0.12
L55M (rs 854560)	Q192R (rs 662)	0.25

### Association of PON1 haplotypes with CAD

On haplotype analysis we observed 32 different combinations of these coding and promoter polymorphisms (Table S1). The frequencies of haplotypes L-T-G-Q-C and L-T-G-R-G (each with four variant and one wild allele) was significantly higher in CAD patients and they were found to be associated with higher risk of CAD with ORs being 3.25 (95% CI: 1.72–6.16) and 2.82 (95% CI: 1.01–7.80) respectively. Frequencies of haplotypes L-C-A-Q-G (with one variant and four wild alleles) (OR = 0.16; 95% CI: 0.08–0.31), L-T-A-Q-G (with two variant and three wild alleles) (OR = 0.51; 95% CI: 0.27–0.96), and M-C-A-Q-G (with all wild alleles) (OR = 0.16; 95% CI: 0.03–0.76) were significantly lower in patients as compared to the controls.

## Discussion

The susceptibility of developing CAD in Asian Indians is 3–4 times higher than Caucasians, 6-times higher than Chinese, and 20-times higher than Japanese [28], [29] and they tend to develop it at a younger age [30], [31]. There are several studies which have investigated the relationship between genetic variability of PON1 and risk of disease. Unfortunately, most of these studies have looked for association of PON1 SNPs with susceptibility and have ignored the more important factor, serum PONase activity [19], [32]. Differences in PON1 activity between populations of the same ethnic group are well known [6], which, if not taken into account, could have affected the results of case-control studies. However, PON1 activity alone is not a good measure of risk of disease as activity is markedly affected by the Q192R polymorphism. Mackness et al. [33] with other PON1 investigators [15], [34] strongly suggest that all further case-control studies should include measurement of the enzyme and its genetic polymorphisms. In this case-control study, we examined both PON1 activity and PON1 polymorphisms. In addition, we also investigated the effect of polymorphisms on serum PON1 activity and lipid levels.

Previous studies that have investigated the relationship between PON1- Q192R polymorphism and CAD have produced inconsistent results. Wheeler et al. [32] in a meta analysis of 35 studies showed that 192R variant was associated with increased risk of CAD with relative risk being 1.12 (95%CI: 1.07–1.16). Mackness et al. [33] in another meta-analysis observed an increased frequency of the PON1 192R allele in CAD patients. However, Schmidt et al. [20] in his study failed to find such an association though they observed that atherosclerotic lesions were more common in RR genotype than QR or QQ genotype. In this study we found that 192R allele frequency was significantly higher in patients and 192QR and 192RR variants were associated with increased risk of CAD with risk being 2.73 (95% CI: 1.57–4.72) and 16.24 (95% CI: 6.41–41.14) respectively, after adjusting all conventional risk factors. Several studies have shown that there are racial differences in association between Q192R polymorphism and CAD risk. Sanghera et al. [35] found that Q192R polymorphism was associated with CAD in Singapore Indians, but not in Singapore Chinese. Zama et al. [36] observed a positive association of Q192R polymorphism in CAD patients in Japanese. Ko et al. [37] in Taiwan population, compared the Q192R genotype distribution in CAD patients with age and sex-matched controls and found no significant difference in the two groups. Similarly Suehiro et al. [38] observed no association in Japanese subjects.

We found lower PON1 activity towards paraoxon (PONase) substrate in CAD patients as compared to the controls, with results being similar to prospective epidemiological Caperhilly [39] study. The low PONase activity is a predictive risk factor for CAD, independent of all other established risk factors such as age, sex, smoking, alcohol and HDL-C levels (Multiple linear regression analysis). These findings indicate that PONase activity plays an important role in the pathogenesis of CAD. However, PONase activity was significantly affected by Q192R polymorphism. It was significantly higher in RR homozygote and lower in QQ homozygote, in both groups. This observation is similar to other studies [40], [41].

PON1 activity is not affected towards phenylacetate (AREase) substrate across Q192R polymorphism [16]. Richter et al. [9] have stated that measurement of AREase activity of PON1 or determination of PON1 protein levels by ELISA are the minimum measures that should be carried out in any epidemiological study. In this study, we observed that AREase activity was significantly lower in patients and was not affected by the Q192R polymorphism and can be considered a surrogate measure of PON1 levels across Q192R genotypes. This has been used in a number of previous studies [9], [16], [17], [42].

Fewer studies have been conducted into the relationship between the PON-L55M polymorphism and CAD but again with inconsistent results [43]. Blatter et al. [44] and Mackness et al. [45] found that L55M polymorphism had significant effect on PON1 activity independent of Q192R polymorphism. We could not observe any significant effect of L55M polymorphisms with CAD and PON1 activity. This is unlike the study by Schmidt et al. [20] who observed an independent association of CAD with L55M polymorphism in Austrian CAD patients.

To the best of our knowledge, not much information is available in literature in terms of PON1 promoter polymorphisms, PON1 activity and CAD risk. We found that PONase activity was significantly higher in wild AA (−162 site), GG (−909 site) and CC (−108C/T site) homozygotes. It increased in the order of the GG<AG<AA genotypes within the −162 A/G polymorphism, CC<CG<GG genotypes in −909G/C polymorphism and TT<CT<CC genotypes within −108C/T polymorphisms in patients and controls. These observations are similar to other studies [46], [47], [48], [49]. In-vitro expression studies reveal that −108C/T polymorphism has a significant effect on expression of PON1 gene as −108C/T polymorphism lies within GG*C*GGG consensus sequence, which is the binding site for the sp1 transcription factor [50]. Najafi et al. [24] reported an association between −108C/T polymorphism and CAD. Leviev et al. [51] observed that −108CC genotype protected against the risk of CAD in patients aged 60 or younger (OR: 0.60; 95% CI: 0.37–0.90) but not in older patients. In our study we observed that −108 C/T polymorphism was not associated with CAD risk after adjusting for conventional risk factors. −909GC (OR: 1.80; 95% CI: 1.24–2.59) and −909CC (OR: 3.03; 95% CI: 1.96–4.68) genotypes of −909G/C polymorphism were associated with CAD risk. Significantly higher distribution of −909C allele in CAD patients (OR: 2.15; 95% CI: 1.53–3.03) was observed as compared to controls. The −162AG (OR: 2.12; 95% CI: 1.41–3.12) and −162GG genotypes (OR: 3.43; 95% CI: 2.19–5.38) of −162A/G polymorphism were also significantly higher in CAD patients. However on multiple logistic regression analysis, only −162GG genotype was independently associated with increased risk of CAD after adjustment for conventional risk factors for CAD. Our study showed that among the studied polymorphisms of PON1 gene, only coding Q192R and promoter −162A/G polymorphisms were independent genetic markers for CAD.

AREase activity was not affected by any of the promoter polymorphisms in any group. These results indicate that AREase activity is a better enzymatic test for examining the association between PON1 activity and disease, especially when genotyping cannot be done. In some studies linkage disequilibrium has been observed between PON1 polymorphism at position 55 with that at position 192 [52]. The −108C/T and −909G/C have been observed to be in linkage disequilibrium with each other. However, no linkage disequilibrium has been observed between PON1 192 polymorphism and −108C/T and −909G/C polymorphisms [53]. In our study we found no linkage disequilibrium in studied SNPs with D' value ranging from 0.03 to 0.25 indicating individual SNPs have an independent association with disease. Our results are similar to that of Jarvik et al. [54] who also did not observe any linkage disequilibrium across the PON1 gene SNPs.

Determination of haplotypes is gaining attention because multiple linked SNPs have the potential to provide significantly more power to genetic analysis than individual SNPs [55]. Information is lacking regarding PON1 haplotypes and CAD risk. We determined haplotype frequencies of coding and promoter polymorphisms by using PHASE software to impute PON1 haplotypes based on genotype information. We observed that L-T-G-Q-C (carrying 4 variant and 1 wild type allele) and L-T-G-R-G (carrying 4 variant and 1 wild type allele) haplotypes were associated with 3.2 and 2.8 fold increase in the risk of CAD. Haplotypes M-C-A-Q-G (carrying all wild type allele), L-T-A-Q-G (carrying 2 variant and 3 wild type allele) and L-C-A-Q-G (carrying 1 variant and 4 wild type allele) were more prevalent in controls with 0.16, 0.51 and 0.16 odds ratio respectively and could be protective of CAD.

In a study of the Hutterite Brethren, a North American population isolated by religious belief, the PON1 genotype was significantly associated with variation in the concentration of HDL-C, LDL-C, TG and apo-B [56]. In them, homozygotes (low-activity variant) of PON1 had significantly lower TG level, LDL-C and apo-B than heterozygotes and homozygotes for the high-activity variant. However we could not find any major effect of PON1 genotypes on lipid levels both in controls and patients.

In conclusion CAD patients had lower PONase and AREase activities as compared to the controls. The coding Q192R, promoter −162A/G polymorphisms and L-T-G-Q-C and L-T-G-R-G haplotypes are all independently associated with CAD.

### Strength and Limitations

The present study has several strengths and limitations that need to be addressed briefly. The strengths include the use of a pure Punjabi population from the North-West region of India to eliminate false positive results due to population stratification. Angiographical profiles of all subjects were evaluated. Regarding limitations, we could not stop statins in CAD patients due to ethical reasons. As statins are known to influence PON1 activity [57], we cannot rule out that they may be having some role in altering PON1 activity in CAD patients. We derived PON1 levels across Q192R genotypes indirectly from AREase activity. Ideal would have been measure it directly by ELISA. However, as it was suggested by several investigators that AREase activity can be used as measure of PON1 levels [9], [16], [17], [58] we used this. In addition, identifying significant associations of genetic variants with complex qualitative trait, such as CAD may demand a larger sample size than for quantitative traits to arrive at worthwhile conclusion. Further studies may be required with more numbers as our study was limited by relatively small groups.

## Materials and Methods

### Subject Selection

Between August 2007 and September 2009, 350 North-West Indian Punjabi's with angiographically proven CAD at Nehru hospital , Postgraduate Institute of Medical Education and Research, Chandigarh (India) were included in the study. Patients with diabetes mellitus, cirrhosis of liver, COPD, HIV infection, malnutrition and acute myocardial infarction were excluded from the study. We also excluded those patients who had undergone a recent coronary intervention i.e. angioplasty or bypass. The disease severity was determined by counting the number of affected three major epicardial coronary arteries with significant stenosis (≥70%). The angiogram was assessed by at least two cardiologists who were unaware of the patient's status before inclusion in the study. In addition, relevant history was recorded regarding risk factors for CAD such as smoking, alcohol consumption, presence or absence of CAD among first degree relatives (parents and siblings) and the age of onset and medical treatment. Due to ethical reasons it was not possible to stop treatment including statins in patients. The dose of atorvastatin varied from 20–80 mg/day with majority on 40 mg. In the control group, 300 North-West Indian Punjabi's who were attending a general medical clinic for health check up and were found to be healthy were included.

### Ethics Statement

All the subjects were provided with the study protocol and an informed written consent was obtained. The study was approved by the institute (Postgraduate Institute of Medical Education and Research) ethics committee.

### Biochemical analysis

Venous blood sample was collected from both controls and CAD patients between 8.30 and 9.30 AM after an overnight fast. The serum was isolated by low-speed centrifugation and enzymatic assays were carried out. The lipid profile (HDL-C, LDL-C, TG and TC) was measured by enzymatic method using autoanalyzer (Hitachi modular P) and Roche diagnostic kits. The PON1 activity and level were measured by using paraoxon and phenylacetate (Sigma Chem,USA) as substrates respectively with activity was measured by a modification of method described by Mackness et al. [6] and level by Eckerson's method [12].

### Genotyping

The DNA was extracted from the blood cells by method described by Daly et al. [59] and was stored at −20°C till genotyping. A total of 5 SNP's of PON1 gene (Accession No. AC004022) were selected for the present study. Coding Q192R [rs662] and L55M [rs854560] genotyping was carried out by PCR amplification and restriction digestion [60]. The promoter −909G/C (rs 854572) and −108C/T (rs 705379) SNP's were genotyped by multiplex PCR whereas −162A/G (rs 705381) SNP, genotyping was carried out by allele specific oligonucleotide-PCR [61]. The primers were designed using PRIMER 3 software. Details of primer sequences, PCR conditions and product size are shown in Table S2. The PCR products were run on 2% agarose gel and were visualized by ethidium bromide staining. The PCR reagents were purchased from Fermentas, Lithuania where as primers were obtained from Operon, USA. For detailed description of this section please see Text S1.

### Statistical analysis

Statistical analysis was carried out using SPSS (Version 17.0) software. All the study variables are shown as mean ± standard deviation or median (range) depending on the shape of the distribution curve. Student's t-test or Mann-Whitney U test was used to compare the data between controls and CAD patients. The demographic parameters were correlated with CAD by univariate logistic regression analysis, and followed up with multiple logistic regression analysis for significant variables. A multiple linear regression analysis was undertaken to determine the independence of PON1 activity from other variables.

The genotype and allele frequencies for each polymorphism were stratified for homozygote wild, heterozygote and homozygote variant type of the respective allelic variant and Pearson's χ^2^ test was used for comparison between groups. The genotypic association with CAD and their odds ratio (OR) with 95% confidence interval was estimated by binary logistic regression, where homozygous wild type was kept as a reference. The effect of genetic variants on CAD susceptibility was determined by multiple logistic regression analysis after adjusting the conventional risk factors. Power of the sample size was calculated using the PAWE software [62]. P values were subjected to Boneferroni's correction and considered significant when P<0.05. The study had more than 80% statistical power to detect an association. Linkage disequilibrium for PON1 polymorphisms was measured using Haploview (Version 4.2) software (http://www.broad.mit.edu/mpg/haploview/contact.php) and was expressed in terms of D'. The haplotype frequency was determined by PHASE (Version 2.1) software (http://stephenslab.uchicago.edu/software.html). Fisher's exact test was used to find differences in haplotype frequency between CAD patients and controls. A p value≤0.05 was considered significant.

## Supporting Information

Table S1 The haplotypes frequency distribution in Controls and CAD patients for coding (Q192R, L55M) and promoter (−909G/C, −162A/G, −108C/T) SNPs of PON1 gene.
(DOC)

Table S2 PCR conditions used in genotyping of promoter SNPs of PON1 gene.
(DOC)

Text S1 (DOCX)

## References

[1] Navab M, Ananthramaiah GM, Reddy ST, Van Lenten BJ, Ansell BJ (2004). The oxidation hypothesis of atherogenesis: the role of oxidized phospholipids and HDL.. J Lipid Res.

[2] Mackness B, Durrington PN, Mackness MI (2002). The paraoxonase gene family and coronary heart disease.. Curr Opin Lipidol.

[3] Cao H, Girard-Globa A, Berthezene F, Moulin P (1999). Paraoxonase protection of LDL against peroxidation is independent of its esterase activity towards paraoxon and is unaffected by the Q→R genetic polymorphism.. J Lipid Res.

[4] Shih DM, Gu L, Xia YR, Navab M, Li WF (1998). Mice lacking serum paraoxonase are susceptible to organophosphate toxicity and atherosclerosis.. Nature.

[5] Shih DM, Xia YR, Wang XP, Miller E, Castellani LW (2000). Combined serum paraoxonase knockout/apolipoprotein E knockout mice exhibit increased lipoprotein oxidation and atherosclerosis.. J Biol Chem.

[6] MacKness B, Mackness MI, Durrington PN, Arrol S, Evans AE (2000). Paraoxonase activity in two healthy populations with differing rates of coronary heart disease.. Eur J Clin Invest.

[7] McElveen J, Mackness MI, Colley CM, Peard T, Warner S (1986). Distribution of paraoxon hydrolytic activity in the serum of patients after myocardial infarction.. Clin Chem.

[8] Mackness MI, Harty D, Bhatnagar D, Winocour PH, Arrol S (1991). Serum paraoxonase activity in familial hypercholesterolaemia and insulin-dependent diabetes mellitus.. Atherosclerosis.

[9] Richter RJ, Jarvik GP, Furlong CE (2010). Paraoxonase 1 status as a risk factor for disease or exposure.. Adv Exp Med Biol.

[10] Aharoni A, Gaidukov L, Khersonsky O, Mc QGS, Roodveldt C (2005). The ‘evolvability’ of promiscuous protein functions.. Nat Genet.

[11] Furlong CE, Richter RJ, Chapline C, Crabb JW (1991). Purification of rabbit and human serum paraoxonase.. Biochemistry.

[12] Gan KN, Smolen A, Eckerson HW, La Du BN (1991). Purification of human serum paraoxonase/arylesterase. Evidence for one esterase catalyzing both activities.. Drug Metab Dispos.

[13] Sorenson RC, Primo-Parmo SL, Kuo CL, Adkins S, Lockridge O (1995). Reconsideration of the catalytic center and mechanism of mammalian paraoxonase/arylesterase.. Proc Natl Acad Sci U S A.

[14] Davies HG, Richter RJ, Keifer M, Broomfield CA, Sowalla J (1996). The effect of the human serum paraoxonase polymorphism is reversed with diazoxon, soman and sarin.. Nat Genet.

[15] Richter RJ, Furlong CE (1999). Determination of paraoxonase (PON1) status requires more than genotyping.. Pharmacogenetics.

[16] Furlong CE, Holland N, Richter RJ, Bradman A, Ho A (2006). PON1 status of farmworker mothers and children as a predictor of organophosphate sensitivity.. Pharmacogenet Genomics.

[17] Richter RJ, Jarvik GP, Furlong CE (2008). Determination of paraoxonase 1 status without the use of toxic organophosphate substrates.. Circ Cardiovasc Genet.

[18] Blatter Garin MC, Abbott C, Messmer S, Mackness M, Durrington P (1994). Quantification of human serum paraoxonase by enzyme-linked immunoassay: population differences in protein concentrations.. Biochem J.

[19] Ruiz J, Blanche H, James RW, Garin MC, Vaisse C (1995). Gln-Arg192 polymorphism of paraoxonase and coronary heart disease in type 2 diabetes.. Lancet.

[20] Schmidt H, Schmidt R, Niederkorn K, Gradert A, Schumacher M (1998). Paraoxonase PON1 polymorphism leu-Met54 is associated with carotid atherosclerosis: results of the Austrian Stroke Prevention Study.. Stroke.

[21] Antikainen M, Murtomaki S, Syvanne M, Pahlman R, Tahvanainen E (1996). The Gln-Arg191 polymorphism of the human paraoxonase gene (HUMPONA) is not associated with the risk of coronary artery disease in Finns.. J Clin Invest.

[22] Arca M, Ombres D, Montali A, Campagna F, Mangieri E (2002). PON1 L55M polymorphism is not a predictor of coronary atherosclerosis either alone or in combination with Q192R polymorphism in an Italian population.. Eur J Clin Invest.

[23] Ombres D, Pannitteri G, Montali A, Candeloro A, Seccareccia F (1998). The gln-Arg192 polymorphism of human paraoxonase gene is not associated with coronary artery disease in italian patients.. Arterioscler Thromb Vasc Biol.

[24] Najafi M, Gohari LH, Firoozrai M (2009). Paraoxonase 1 gene promoter polymorphisms are associated with the extent of stenosis in coronary arteries.. Thromb Res.

[25] Rose HA (1970). A glossary of the tribes and castes of Punjab and northwest frontier province. In census report language Department.. Punjab.

[26] Singh S, Verma M, Nain CK, Leelamma CO, Goel RC (1999). Paraoxonase (PON1) polymorphism & its relation with lipids in north west Indian Punjabis.. Indian J Med Res.

[27] Singh S, Venketesh S, Verma JS, Verma M, Lellamma CO (2007). Paraoxonase (PON1) activity in north west Indian Punjabis with coronary artery disease & type 2 diabetes mellitus.. Indian J Med Res.

[28] Enas EA, Yusuf S, Sharma S (1998). Coronary artery disease in South Asians. Second meeting of the International Working Group. 16 March 1997, Anaheim, California.. Indian Heart J.

[29] Enas EA, Garg A, Davidson MA, Nair VM, Huet BA (1996). Coronary heart disease and its risk factors in first-generation immigrant Asian Indians to the United States of America.. Indian Heart J.

[30] Janus ED, Postiglione A, Singh RB, Lewis B (1996). The modernization of Asia. Implications for coronary heart disease. Council on Arteriosclerosis of the International Society and Federation of Cardiology.. Circulation.

[31] McKeigue PM, Ferrie JE, Pierpoint T, Marmot MG (1993). Association of early-onset coronary heart disease in South Asian men with glucose intolerance and hyperinsulinemia.. Circulation.

[32] Wheeler JG, Keavney BD, Watkins H, Collins R, Danesh J (2004). Four paraoxonase gene polymorphisms in 11212 cases of coronary heart disease and 12786 controls: meta-analysis of 43 studies.. Lancet.

[33] Mackness B, Davies GK, Turkie W, Lee E, Roberts DH (2001). Paraoxonase status in coronary heart disease: are activity and concentration more important than genotype?. Arterioscler Thromb Vasc Biol.

[34] Jarvik GP, Rozek LS, Brophy VH, Hatsukami TS, Richter RJ (2000). Paraoxonase (PON1) phenotype is a better predictor of vascular disease than is PON1(192) or PON1(55) genotype.. Arterioscler Thromb Vasc Biol.

[35] Sanghera DK, Saha N, Aston CE, Kamboh MI (1997). Genetic polymorphism of paraoxonase and the risk of coronary heart disease.. Arterioscler Thromb Vasc Biol.

[36] Zama T, Murata M, Matsubara Y, Kawano K, Aoki N (1997). A 192Arg variant of the human paraoxonase (HUMPONA) gene polymorphism is associated with an increased risk for coronary artery disease in the Japanese.. Arterioscler Thromb Vasc Biol.

[37] Ko YL, Ko YS, Wang SM, Hsu LA, Chang CJ (1998). The Gln-Arg 191 polymorphism of the human paraoxonase gene is not associated with the risk of coronary artery disease among Chinese in Taiwan.. Atherosclerosis.

[38] Suehiro T, Nakauchi Y, Yamamoto M, Arii K, Itoh H (1996). Paraoxonase gene polymorphism in Japanese subjects with coronary heart disease.. Int J Cardiol.

[39] Mackness B, Durrington P, McElduff P, Yarnell J, Azam N (2003). Low paraoxonase activity predicts coronary events in the Caerphilly Prospective Study.. Circulation.

[40] Nevin DN, Zambon A, Furlong CE, Richter RJ, Humbert R (1996). Paraoxonase genotypes, lipoprotein lipase activity, and HDL.. Arterioscler Thromb Vasc Biol.

[41] Mackness B, Mackness MI, Arrol S, Turkie W, Julier K (1998). Serum paraoxonase (PON1) 55 and 192 polymorphism and paraoxonase activity and concentration in non-insulin dependent diabetes mellitus.. Atherosclerosis.

[42] Berkowitz GS, Wetmur JG, Birman-Deych E, Obel J, Lapinski RH (2004). In utero pesticide exposure, maternal paraoxonase activity, and head circumference.. Environ Health Perspect.

[43] Mackness MI, Durrington PN, Mackness B (2000). How high-density lipoprotein protects against the effects of lipid peroxidation.. Curr Opin Lipidol.

[44] Blatter MC, James RW, Messmer S, Barja F, Pometta D (1993). Identification of a distinct human high-density lipoprotein subspecies defined by a lipoprotein-associated protein, K-45. Identity of K-45 with paraoxonase.. Eur J Biochem.

[45] Mackness B, Mackness MI, Arrol S, Turkie W, Durrington PN (1997). Effect of the molecular polymorphisms of human paraoxonase (PON1) on the rate of hydrolysis of paraoxon.. Br J Pharmacol.

[46] Brophy VH, Hastings MD, Clendenning JB, Richter RJ, Jarvik GP (2001). Polymorphisms in the human paraoxonase (PON1) promoter.. Pharmacogenetics.

[47] James RW, Leviev I, Ruiz J, Passa P, Froguel P (2000). Promoter polymorphism T(−107)C of the paraoxonase PON1 gene is a risk factor for coronary heart disease in type 2 diabetic patients.. Diabetes.

[48] Deakin S, Leviev I, Brulhart-Meynet MC, James RW (2003). Paraoxonase-1 promoter haplotypes and serum paraoxonase: a predominant role for polymorphic position - 107, implicating the Sp1 transcription factor.. Biochem J.

[49] Leviev I, James RW (2000). Promoter polymorphisms of human paraoxonase PON1 gene and serum paraoxonase activities and concentrations.. Arterioscler Thromb Vasc Biol.

[50] Saikawa Y, Price K, Hance KW, Chen TY, Elwood PC (1995). Structural and functional analysis of the human KB cell folate receptor gene P4 promoter: cooperation of three clustered Sp1-binding sites with initiator region for basal promoter activity.. Biochemistry.

[51] Leviev I, Righetti A, James RW (2001). Paraoxonase promoter polymorphism T(−107)C and relative paraoxonase deficiency as determinants of risk of coronary artery disease.. J Mol Med.

[52] Hernandez AF, Mackness B, Rodrigo L, Lopez O, Pla A (2003). Paraoxonase activity and genetic polymorphisms in greenhouse workers with long term pesticide exposure.. Hum Exp Toxicol.

[53] Suehiro T, Nakamura T, Inoue M, Shiinoki T, Ikeda Y (2000). A polymorphism upstream from the human paraoxonase (PON1) gene and its association with PON1 expression.. Atherosclerosis.

[54] Jarvik GP, Hatsukami TS, Carlson C, Richter RJ, Jampsa R (2003). Paraoxonase activity, but not haplotype utilizing the linkage disequilibrium structure, predicts vascular disease.. Arterioscler Thromb Vasc Biol.

[55] Cambien F, Malcolm S, Goodship J (2001). Genes in population.. Genotype to Phenotype.

[56] Hegele RA, Brunt JH, Connelly PW (1995). A polymorphism of the paraoxonase gene associated with variation in plasma lipoproteins in a genetic isolate.. Arterioscler Thromb Vasc Biol.

[57] Gouedard C, Koum-Besson N, Barouki R, Morel Y (2003). Opposite regulation of the human paraoxonase-1 gene PON-1 by fenofibrate and statins.. Mol Pharmacol.

[58] Berkowitz GS, Wolff MS, Lapinski RH, Todd AC (2004). Prospective study of blood and tibia lead in women undergoing surgical menopause.. Environ Health Perspect.

[59] Daly AK, Steen VM, Fairbrother KS, Idle JR (1996). CYP2D6 multiallelism.. Methods Enzymol.

[60] Eckerson HW, Wyte CM, La Du BN (1983). The human serum paraoxonase/arylesterase polymorphism.. Am J Hum Genet.

[61] Rompler H, Dear PH, Krause J, Meyer M, Rohland N (2006). Multiplex amplification of ancient DNA.. Nat Protoc.

[62] Gordon D, Finch SJ, Nothnagel M, Ott J (2002). Power and sample size calculations for case-control genetic association tests when errors are present: application to single nucleotide polymorphisms.. Hum Hered.

