# Supplementary material for: Paraoxonase 1 (PON1) Polymorphisms, Haplotypes and Activity in Predicting CAD Risk in North-West Indian Punjabis
Source: PLoS One. 2011 May 24;6(5):e17805. doi: 10.1371/journal.pone.0017805 (PMC3101202; doi:10.1371/journal.pone.0017805)
Supplement: Table S1 — The haplotypes frequency distribution in Controls and CAD patients for coding (Q192R, L55M) and promoter (−909G/C, −162A/G, −108C/T) SNPs of PON1 gene. (DOC) [file pone.0017805.s001.doc]

**Supplemental Table** 1

| **Haplotypes*** | **Control** | **CAD** | **P value** | **OR** |
| --- | --- | --- | --- | --- |
| LCAQC | 0.063 | 0.032 | 0.06 | 0.47 (0.22-1.02) |
| LCAQG | 0.166 | 0.031 | 0.00001 | 0.16 (0.08-0.31) |
| LCARC | 0.019 | 0.039 | 0.17 | 2.04 (0.77-5.38) |
| LCARG | 0.036 | 0.050 | 0.44 | 1.42 (0.66-3.06) |
| LCGQC | 0.049 | 0.060 | 0.61 | 1.21 (0.61-2.39) |
| LCGQG | 0.073 | 0.055 | 0.33 | 0.72 (0.38-1.36) |
| LCGRC | 0.031 | 0.046 | 0.31 | 1.54 (0.67-3.58) |
| LCGRG | 0.038 | 0.040 | 1 | 1 (0.45-2.19) |
| LTAQC | 0.047 | 0.073 | 0.18 | 1.63 (0.83-3.20) |
| LTAQG | 0.090 | 0.047 | 0.04 | 0.51 (0.27-0.96) |
| LTARC | 0.014 | 0.034 | 0.12 | 2.62 (0.83-8,.23) |
| LTARG | 0.015 | 0.031 | 0.31 | 1.91 (0.65-5.57) |
| LTGQC | 0.045 | 0.129 | 0.0001 | 3.25 (1.72-6.16) |
| LTGQG | 0.060 | 0.079 | 0.35 | 1.36 (0.73-2.51) |
| LTGRC | 0.048 | 0.052 | 0.85 | 1.10 (0.54-2.26) |
| LTGRG | 0.017 | 0.046 | 0.04 | 2.82 (1.01-7.80) |
| MCAQC | 0.015 | 0.011 | 1 | 0.85 (0.21-3.45) |
| MCAQG | 0.033 | 0.007 | 0.01 | 0.16 (0.03-0.76) |
| MCARC | 0.005 | 0.006 | 1 | 1.71 (0.15-19.04) |
| MCARG | 0.007 | 0.009 | 1 | 1.28 (0.21-7.76) |
| MCGQC | 0.010 | 0.017 | 0.51 | 1.72 (0.42-6.96) |
| MCGQG | 0.009 | 0.012 | 1 | 1.14 (0.25-5.15) |
| MCGRC | 0.004 | 0.005 | 1 | 1.71 (0.15-19.04) |
| MCGRG | 0.009 | 0.009 | 1 | 0.85 (0.17-4.27) |
| MTAQC | 0.014 | 0.011 | 1 | 0.85 (0.21-3.45) |
| MTAQG | 0.026 | 0.009 | 0.12 | 0.31 (0.08-1.20) |
| MTARC | 0.004 | 0.004 | 1 | 0.85 (0.05-13.75) |
| MTARG | 0.004 | 0.008 | 0.62 | 2.58 (0.26-24.98) |
| MTGQC | 0.021 | 0.015 | 0.76 | 0.71 (0.21-2.35) |
| MTGQG | 0.021 | 0.015 | 0.76 | 0.71 (0.21-2.35) |
| MTGRC | 0.005 | 0.007 | 0.62 | 2.58 (0.26-24.98) |
| MTGRG | 0.002 | 0.007 | 1 | 1.71 (0.15-19.04) |

* Order of SNPs is: L55M (rs 854560), -108C/T (rs 705379), -162A/G (rs 705381), Q192R
(rs 662) and -909G/C (rs 854572).
