# Supplementary material for: Paraoxonase 1 (PON1) Polymorphisms, Haplotypes and Activity in Predicting CAD Risk in North-West Indian Punjabis
Source: PLoS One. 2011 May 24;6(5):e17805. doi: 10.1371/journal.pone.0017805 (PMC3101202; doi:10.1371/journal.pone.0017805)
Supplement: Table S2 — PCR conditions used in genotyping of promoter SNPs of PON1 gene. (DOC) [file pone.0017805.s002.doc]

**Supplemental Table 2:**

| SNP’s (rs) | Primers | Amplicon  (bp) | Annealing temp. (^0^C/)/[MgCl_2_](mmol) | Allele size |
| --- | --- | --- | --- | --- |
| -108(C/T)  [rs705379] | *FW: 5'-GACCCGGCGGGGAGGGG**C**-3' | 417 | 60/2.0 | Multiplex PCR |
|  | **FV: 5'-GACCCGGCGGGGAGGGG**T**-3' |  |  | C = 417, T = 417 |
|  | ***RC1: 5'-TTGCCTCCTTATCCCATGTCTC-3' |  |  |  |
| -909(G/C)  [rs854572] | FW: 5'-CAGCAGACAGCAGAGAAGAGA**G**-3' | 286 | 60/2.0 | G = 286,C = 286 |
|  | FV: 5'-CAGCAGACAGCAGAGAAGAGA**C**-3' |  |  |  |
|  | RC2: 5'-ACAAAACGTCTTCCTCAAGCTTAC-3' |  |  |  |
| -162(A/G)  [rs705381] | FW: 5'-TGGGGGCTGACCGCAAGCC**A**-3' | 472 | 60/1.5 | ASO PCR |
|  | FV: 5'-TGGGGGCTGACCGCAAGCC**G**-3' |  |  | A = 472,G = 472 |
|  | RC: 5'-TTGCCTCCTTATCCCATGTCTC-3' |  |  |  |

*FW= forward wild, **FV= forward variant, ***RC= reverse common

ASO (allele specific oligonucleotide)
