# Supplementary material for: Paraoxonase 1 (PON1) Polymorphisms, Haplotypes and Activity in Predicting CAD Risk in North-West Indian Punjabis
Source: PLoS One. 2011 May 24;6(5):e17805. doi: 10.1371/journal.pone.0017805 (PMC3101202; doi:10.1371/journal.pone.0017805)
Supplement: Text S1 — (DOCX) [file pone.0017805.s003.docx]

**“Supplemental Methods”**

**PON1 Q192R and L55M genotyping**

Q192R and L55M genotypes were determined by PCR amplification and restriction digestion. Q192R polymorphism was analyzed using the following primers: FORWARD5’ TATTGTTGCTGTGGGACCTGAG 3’ and REVERSE 5’ CCTGAGAATCTGAGTAAATCCACT 3’. PCR reaction (total volume 25µl) contained 100ng DNA template, 200µM dNTPs, 300nM of each primer, 1.5 mM MgCl_2_ and 1U Taq polymerase (Fermentas). 35 cycles of amplification (3 min at 94^0^C followed by 30 sec at 95^0^C and 20 sec at specific primers annealing temperature at 60.1^0^C and 50 sec at 72^0^C) with a final extension of 10 min at 72^0^C were performed. PCR products were digested with *AlwI* (New Engald Biolab) and resulted in 66 and 172-bp fragments for the 192R and in a nondigested 238-bp fragment for the 192Q allele.

The PON1 L55M polymorphism was analyzed using the following primers: FORWARD 5’TGAACAGTCGCCAAGAGATG 3’ and REVERSE 5’ CTGGCACACACATTTTCCAC3’ (annealing temperature: 60.3^0^C). PCR products were digested with *NlaIII* (New Engald Biolab) and resulted in 66- and 106-bp fragment for the 55M allele and in non-digested 172-bp fragment for the 55L allele.

**PON1 -909G/C and -108C/T genotyping**

Promoter -909G/C and -108C/T genotyping were carried out by multiplex PCR. In this PCR two tubes were used for each DNA sample. In one tube we used forward wild primers of both SNPs and in another tube forward variant primer were used whereas reverse primer was common in both tubes. PCR reaction (total volume 25µl) contained 100ng DNA template, 200µM dNTPs, 300nM of each primer, 1.5 mM MgCl_2_ and 1U Taq polymerase (Fermentas). 35 cycles of amplification (5 min at 95^0^C followed by 30 sec at 940C and 35 sec at specific primers annealing temperature at 610C and 20 sec at 720C) with a final extension of 10 min at 720C were performed. The PCR products were run on 2% agarose gel and visualized by ethidium bromide staining. PCR products were 286 and 417- bp long for -909G/C and -108C/T polymorphisms respectively. If bands were present in wild lane then correspond to -909GG and -108CC genotype, if present in variant lane correspond to -909CC and -108TT and if present in both lane then correspond to -909GC and -108CT genotypes respectively.

**PON1-162A/G genotyping**

Genotyping at -162A/G site was determined by allele specific oligonucleotide (ASO) PSR. In this PCR allele specific primers were synthesized and two tubes for each sample was prepared. In on tube we added forward wild primer and in another tube forward variant primer was added. Reverse primer was common in both tubes. PCR reaction (total volume 25µl) contained 100ng DNA template, 200µM dNTPs, 300nM of each primer, 2.0 mM MgCl_2_ and 1U Taq polymerase (Fermentas). 35 cycles of amplification (5 min at 950C followed by 30 sec at 940C and 35 sec at specific primers annealing temperature at 610C and 20 sec at 72^0^C) with a final extension of 10 min at 72^0^C were performed. The PCR products were run on 2% agarose gel and visualized by ethidium bromide staining. PCR product was 472 bp long. If band was present in wild lane then correspond to AA genotype, if present in variant lane correspond to GG and if present in both lane then correspond to AG genotypes respectively.
